# The Effect of the Pyrethroid Pesticide Fenpropathrin on the Cardiac Performance of Zebrafish and the Potential Mechanism of Toxicity

**DOI:** 10.3390/biology12091214

**Published:** 2023-09-06

**Authors:** Ferry Saputra, Yu-Heng Lai, Marri Jmelou M. Roldan, Honeymae C. Alos, Charlaine A. Aventurado, Ross D. Vasquez, Chung-Der Hsiao

**Affiliations:** 1 Department of Chemistry, Chung Yuan Christian University, Taoyuan 320314, Taiwan; ferrysaputratj@gmail.com; 2 Department of Bioscience Technology, Chung Yuan Christian University, Taoyuan 320314, Taiwan; 3 Department of Chemistry, Chinese Culture University, Taipei 11114, Taiwan; lyh21@ulive.pccu.edu.tw; 4 The Graduate School, University of Santo Tomas, Manila 1008, Philippines; mmroldan@ust.edu.ph (M.J.M.R.); honeymae.alos.gs@ust.edu.ph (H.C.A.); charlaine.aventurado.gs@ust.edu.ph (C.A.A.); 5 Department of Pharmacy, Faculty of Pharmacy, University of Santo Tomas, Manila 1008, Philippines; 6 Research Center for Natural and Applied Sciences, University of Santo Tomas, Manila 1008, Philippines; 7 Research Center for Aquatic Toxicology and Pharmacology, Chung Yuan Christian University, Taoyuan 320314, Taiwan

**Keywords:** fenpropathrin, zebrafish, cardiovascular toxicity, pesticide

## Abstract

**Simple Summary:**

In recent years, the human population on Earth has increased, leading to a higher demand for food sources. Pesticides have emerged as a breakthrough to enhance crop yields. However, the excessive use of pesticides has raised concerns regarding their potential impact on aquatic animals. Thus, this study aims to assess the potential cardiovascular toxicity of various pesticides, with a specific focus on fenpropathrin. When compared to other pesticides, fenpropathrin causes cardiomegaly, an increased heart rate, a significant rise in blood flow velocity, and an elevated metabolism rate. Furthermore, it has been found to bind to several ion channels, inducing alterations in cardiovascular-related markers. These findings indicate that fenpropathrin induces cardiotoxicity in the zebrafish larvae model.

**Abstract:**

Fenpropathrin, a pyrethroid insecticide, has been widely used for many years in agricultural fields. It works by disturbing the voltage-gated sodium channel, leading to paralysis and the death of the target animal. While past studies have focused on neurodegeneration following fenpropathrin poisoning in humans, relatively few pieces of research have examined its effect on other peripheral organs. This study successfully investigated the potential toxicity of fenpropathrin on the cardiovascular system using zebrafish as an animal model. Zebrafish larvae exposed to varying doses of fenpropathrin underwent an evaluation of cardiac physiology by measuring the heart rate, stroke volume, cardiac output, and shortening fraction. The blood flow velocity and the dorsal aorta diameter were also measured to assess the impact of fenpropathrin exposure on the vascular system. Furthermore, molecular docking was performed to evaluate the pesticide binding affinity to various proteins associated with the cardiovascular system, revealing the potential mechanism of the fenpropathrin cardiotoxic effect. The findings demonstrated a significant dose-dependent increase in the heart rate stroke volume, cardiac output, shortening fraction, and ejection fraction of zebrafish larvae after 24 h of acute treatment with fenpropathrin. Additionally, zebrafish treated at a concentration of 1 ppm exhibited significantly larger blood vessels in diameter and an increased blood flow velocity compared to the control group. According to molecular docking, fenpropathrin showed a high affinity for various voltage-gated sodium channels like scn1lab, cacna1sb, and clcn3. Finally, from the results, we found that fenpropathrin caused cardiomegaly, which may have been induced by the voltage-gated sodium channel disruption. This study highlights the significant disruption of fenpropathrin in the cardiovascular system and emphasizes the need for further research on the health implications of this pesticide.

## 1. Introduction

As the population of humans rises, food production has to increase. However, issues with pests and other unwelcome species have always put the productivity of crops at risk. In the past, preventive measures like setting traps, weeding, and manually removing pests were performed, but as the amount of land necessary for crop production increased, so did the amount of laborers needed, which decreased efficiency and increased production costs. To combat this issue, pesticides were invented and applied in agricultural fields to reduce or eradicate uncontrollably proliferating organisms in specific locations [1]. These pesticides can be divided into herbicide, insecticide, nematicide, molluscicide, piscicide, avicide, rodenticide, bactericide, microbicide, fungicide, and lampricide categories depending on the target organism [2].

One widely used insecticide is fenpropathrin, which belongs to the pyrethroid group. Like natural pyrethrins, pyrethroid is an organic compound that is naturally produced by pyrethrum flowers. Pyrethroids are divided into two groups based on their chemical structure: type I, which does not have an alpha-cyano group on the phenoxy benzyl moiety, and type II, which does. It causes mortality and paralysis by interfering with voltage-gated sodium channels [3]. It was discovered by a Japanese company in 1971 and commercialized in 1980 as the first light-stable synthetic pyrethroid. Despite being quickly metabolized and eliminated from the body, recent research indicates that it may be hazardous to fish and has long-lasting effects in water [4,5,6,7]. In Taiwan, the government has set the maximum residue limits of fenpropathrin in fruit and vegetables since 2012, and recently this year, the European Food Safety Authority also recommended lowering the maximum tolerated residue level of fenpropathrin in fruits and vegetables [8,9].

It has been reported several times that human exposure to pyrethroid can be dangerous [10,11,12]. A study conducted by Xiong et al. (2016) in 2015 showed that a patient who consumed fish that had been exposed to fenpropathrin every day for six months developed Parkinsonian symptoms. This finding led their research team to investigate the potential neurotoxicity of fenpropathrin to the dopaminergic system. In vitro and in vivo studies support that fenpropathrin poisoning could mimic the pathologic and pathogenetic features of Parkinson’s disease [12]. A subsequent study by Jiao et al. (2020) further demonstrated that the mitochondrial quality control system was disrupted, causing the degeneration of dopaminergic neurons in mouse models [13]. In zebrafish, Yu et al. (2022) reported reduced locomotor activity and suppressed expression of genes associated with neurodevelopment in zebrafish [14]. Nieradko-Iwanicka and Bozecki also noted that fenpropathrin exposure for 28 days increases the expression of antioxidant enzymes in the mouse liver in response to oxidative stress and decreases the activity of antioxidant enzymes in the mouse brain [15,16]. This suggests that fenpropathrin may have severe negative consequences on animals that are not its intended target.

In recent years, zebrafish have emerged as an important animal model for studying aquatic toxicity after researchers found that the toxic profiles in zebrafish are similar to those in mammalian models, which has been confirmed by various studies [17,18,19,20,21,22,23]. Its high reproductive rate and sensitivity to change in water quality make it a popular model for drug testing. Zebrafish also makes an excellent animal model for genetic studies, with around 70% of its genes being homologous to their human counterparts [24]. The transparent body of zebrafish at the larval stage allows researchers to observe the cardiovascular system’s development in zebrafish. Interestingly, the heart-pumping mechanism of zebrafish is nearly identical to that of humans, and several studies have reported that 95% of drugs that lengthen the QT interval in humans also have the same effect on zebrafish [25]. All those traits make zebrafish a good animal model for cardiotoxicity studies.

The focus of this research was to explore the potential cardiotoxicity effects of pesticides in zebrafish larvae and later focus more on fenpropathrin as it shows different kinds of alterations to cardiovascular performance parameters compared with the other pesticides. Research has shown that the effects of fenpropathrin on the nervous system have been extensively studied; however, there has yet to be a follow-up study on its potential cardiotoxicity, although fenpropathrin is known to affect the voltage-gated sodium channel that also localizes in cardiomyocytes [26,27]. A study by Xiong et al. in 2016 reported that fenpropathrin exposure can cause pathological changes in the peripheral organs and heart muscles of mice, and in this study, we further explore the effect of fenpropathrin on the cardiovascular system by assessing the cardiovascular performance and metabolic rate, followed by gene expression analysis [12].

## 2. Material and Method

### 2.1. Animal Husbandry and Exposure

The AB strain zebrafish was obtained from Taiwan Zebrafish Stock Center at Academia Sinica (TZCAS) (Taipei, Taiwan) and maintained in a continuous aerated water system. The temperature was maintained at 26 °C with 10/14 h of dark/light cycle. At night, two adult males and one adult female zebrafish were kept in a breeding chamber overnight to obtained the eggs for further experiments. The separator of the breeding chamber was opened on the following day, and the fertilized eggs were collected after two hours. The eggs were incubated at 28 °C and at 48 h post fertilization (hpf). The eggs were continuously exposed to pesticides by putting them into 5 cm Petri dishes with 10 mL of pesticide with desired concentrations until three days post fertilization (dpf) for the potential cardiovascular toxicity analysis. All protocols and procedures involving zebrafish in this experiment were approved by the Committee for Animal Experimentation of the Chung Yuan Christian University (Approval No. 109001, issue date: 15 January 2020).

### 2.2. Chemical Preparation

Chlorantriniprolle, diuron, fenpropathrin, fipronil, imidacloprid, oxyfluorfen, paraquat, and thiobencarb with >95% purity were purchased from Aladdin Bio-Chem Technology Co., Ltd. Shanghai, China. All of the pesticides were diluted with acetone to create a 10 g/L stock solution and then with ddH_2_O to produce a 100 ppm (*w*/*v*) working solution. For the whole experiment, acetone was used as the solvent control at a concentration of 0.01%. Just before exposure, the stock solution was further diluted with ddH_2_O until the desired concentration was reached.

### 2.3. Acute Toxicity Test

To check that an effective concentration of fenpropathrin was used for analysis, acute toxicity testing on zebrafish larvae was performed in accordance with Section 2 No. 203 of the OECD Guidelines [28]. Eight 24 hpf zebrafish eggs were placed into 48-well plate, and each well was added with one milliliter of desired test concentrations of fenpropathrin (0.01, 0.1, 1, and 10 ppm). Following exposure, the plate was put into an incubator at 26 °C with a 10/14 h dark/light cycle. The mortality rate was documented every 24 h at 48, 72, 96, and 120 hpf, and dead fish were removed at every examination. The experiment was performed in five replicates to ensure the accuracy of data.

### 2.4. Cardiac Performance Analysis

Cardiac performance analysis was performed by analyzing the cardiac physiology parameters, namely stroke volume (SV), cardiac output (CO), shortening fraction (SF), and ejection fraction (EF), and the cardiac rhythm parameters consist of the heart rate (HR) and heart rate variability. Zebrafish larvae at 72 hpf that had been mounted in 3% methylcellulose were used for the recording and analysis. The recording was performed using high-resolution 4K CCD (XP4K8MA, ToupTek, Hangzhou, China) mounted on an upright microscope (EX20, SOPTOP, Taipei, Taiwan). The ventricle chamber was recorded for one minute for each zebrafish with a resolution of 1920 × 1080 at 30 frames per second (fps). The cardiac performance calculation was obtained based on the length of the short axis (Ds) and long axis (DL) of the heart chamber during the diastolic and systolic phases to obtain the end diastolic volume (EDV) and the end systolic volume (ESV) by assuming that the heart chamber had a spheroid shape [29]. The following formula was used to calculate the heart physiology:

SV=EDV−ESV

CO=SV×HR

$$EF\left(\%\right)=\frac{SV}{EDV}\times 100\%$$

$$SF\left(\%\right)=\frac{Ds\left(EDV\right)-Ds\left(ESV\right)}{Ds\left(EDV\right)}\times 100\%$$

Time Series Analyzer V3 Plug-in on ImageJ Software v1.52 (https://imagej.nih.gov/ij/plugins/time-series.html (accessed on 17 March 2023)) obtains the dynamic pixel intensity in the selected region of interest, and the program reports it on a frame-by-frame basis. Based on that information, the interval of each ventricle beat in the video can be calculated to obtain the heart rate according to the protocol developed by Sampurna et al. [30]. Sd1 and sd2 of the ventricle chamber beat interval generated from a point care plot using OriginPro 2019 Software v9.6.5.169 (Originlab Corporation, Northampton, MA, USA) were used to calculate the heartbeat variability. Ten fishes were used for each treatment, and experiments were performed in triplicate.

### 2.5. Vascular Performance Analysis

Vascular performance analysis was performed using the same setting as cardiac performance analysis. However, the recording was performed using a high-speed digital charged coupling device (CCD; AZ Instrument, Taichung City, Taiwan) camera mounted on an inverted microscope (ICX41, Sunny Optical Technology, YuYao, China) that was capable of recording at 200 frames per second (fps) for 10 s. Hoffman modulation contrast objective lens with 40× magnification was used to record the dorsal aorta area. “Trackmate” plugin in ImageJ was then used to calculate the blood flow velocity according to the previous protocol by Santoso et al. [31,32,33], followed by calculating the diameter of the dorsal aorta using ImageJ. Ten fishes were used for each treatment, and experiments were performed in triplicate.

### 2.6. Oxygen Consumption Analysis

Oxygen consumption assessment was performed using a 24-well plate placed in a Sensor Dish Reader (SDR), and the oxygen rate in the plate was recorded from time to time using MicroResp^®^ version 1.0.4 (Loligo Systems, Viborg, Denmark) software. Approximately 23 zebrafish embryos were placed into the wells and exposed to fenpropathrin at various concentrations. In total, 80 µL of the fenpropathrin was put into each well, while leaving the blank well without the larvae. The oxygen consumption rate was continuously observed for 60 min, and the experiment was performed in triplicate.

### 2.7. RT-PCR for Genes Related to Cardiovascular Performance

About 40 live embryos were collected after exposure, and several genes related to cardiovascular development were selected, including the transcription factors *gata4*, *myc6*, *tbx5*, *nkx2.5*, *nppa* (atrial natriuretic peptide), *nppb* (B-type natriuretic peptide), and the myosin heavy chains *amhc* and *vmhc*. Furthermore, gene expression related to zebrafish vascular system development was observed, e.g., *gata1*, *vegfaa*, and *vegfab*. The total RNA was extracted using RNAzol reagent following the protocol provided by the manufacturer. The qRT-PCR was performed using a commercial kit following the manufacturer’s instructions, and the experiment was performed in four replicates per sample (*n* = 40/sample). The expression of cardiovascular- and apoptosis-related genes was normalized with the relative expression of *β-actin* as an internal control. The sequence of each primer used for qRT-PCR is summarized in Table 1.

### 2.8. Molecular Docking Analysis

The 3D structure of fenpropathrin was obtained from PubChem (https://pubchem.ncbi.nlm.nih.gov/ (accessed on 23 March 2023)), while the zebrafish voltage-gated sodium, calcium, and chloride ion channel proteins and protein related to apoptosis were retrieved from the AlphaFold protein structure database (https://alphafold.ebi.ac.uk/ (accessed on 23 March 2023)). To conduct protein–ligand docking, the AutoDock Vina wizard v1.1.2 in Python Prescription (PyRx) virtual screening software was utilized [34]. Moreover, PyRx’s Open Babel was used to prepare the ligand structure, and PyRx’s AutoDock Tool was used to process and prepare the proteins. A grid box was also produced around the target region of the protein to allow the ligands to dock in the binding site. Its size was optimized and is demonstrated in Table 2. The ligand was docked using PyRx’s AutoDock Vina wizard via the Assisted Model Building with Energy Refinement (AMBER) force field as the scoring parameter. The docking poses and ligand interactions were graphically visualized and investigated using the Discovery Studio (DS) 2021 Client (Accelrys Inc., San Diego, CA, USA).

### 2.9. Statistical Analysis

All data were analyzed using GraphPad Prism 8 software (GraphPad Software Inc., La Jolla, CA, USA). Statistical significance was determined by parametric or non-parametric analysis according to data normality and distribution.

## 3. Result

### 3.1. Testing Cardiovascular and Metabolic Performance Alterations after Fenpropathrin Exposure

To evaluate the potential cardiovascular toxicity of pesticides in zebrafish, we conducted a small-scale screening of eight pesticides of chlorantriniprolle, diuron, fenpropathrin, fipronil, imidacloprid, oxyfluorfen, paraquat, and thiobencarb, which are pesticide residues that are often detected in Taiwan. Zebrafish embryos were incubated with different doses of tested chemicals with concentrations of 0.01, 0.1, 1, and 10 ppm, and several important cardiac performance endpoints were measured and compared after 24 h of acute incubation. Among all the tested chemicals, fenpropathrin showed the most significant alterations for cardiac performance and will be selected for conducting the detailed check in this study. Cardiac performance alteration data for other pesticide-exposed larvae are provided in Figures S1–S7 for reference.

**Figure 1:**
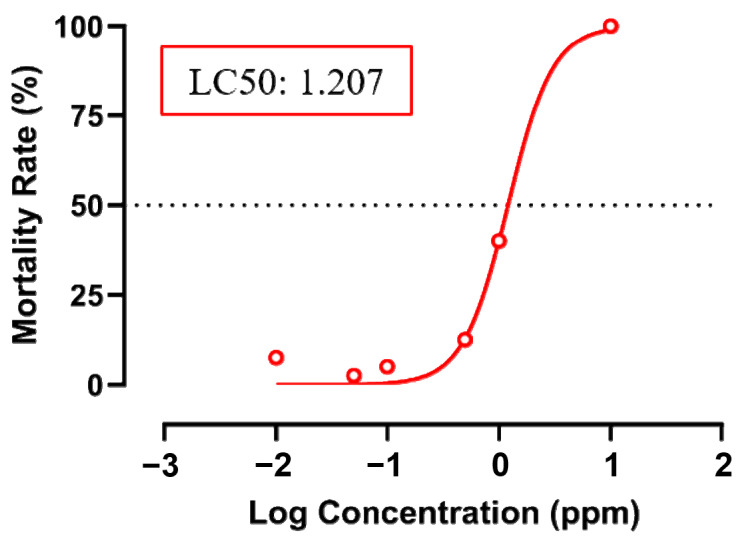
Mortality rate of zebrafish embryos after incubation in several concentrations of fenpropathrin and the LC_50_ value. Red line show the non-linear curve for the LC_50_ calculation.

For fenpropathrin, an acute toxicity exposure test prior to the other followed-up experiment was performed to check the effective concentration. Based on the LC50 experiment, we found out that fenpropathrin at a concentration of 1.207 ppm (*w*/*v*) was able to kill 50% of the zebrafish (LC_50_), which is considered moderately toxic according to the US Environmental Protection Agency (Figure 1) [35]. Based on this, a further experiment was performed at sub-lethal concentrations obtained from the LC_50_ data.

**Figure 2:**
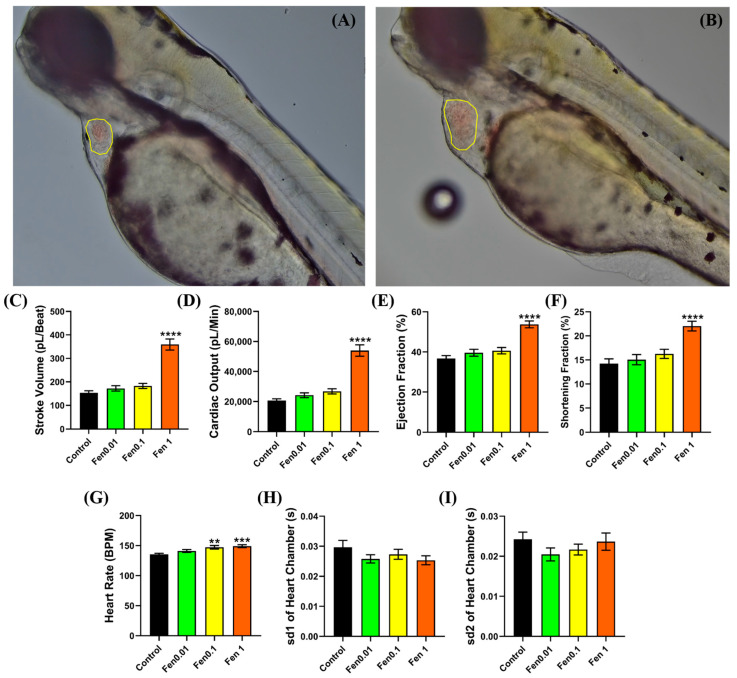
Ventricle chamber of the control group (**A**) and 1-ppm-treated group (**B**) after 24 h of incubation in fenpropathrin at the end of the diastolic phase. Yellow lines show the outline of the ventricle chamber. Cardiac performance parameters after 24 h of acute incubation in different concentrations of fenpropathrin from 0.01 to 1 ppm (**C**–**I**). The data were presented as mean ± statistical error mean (SEM), and the statistical significance was calculated using Ordinary One-Way ANOVA with Dunnet multiple comparison test. (** *p* < 0.01, *** *p* < 0.001, **** *p* < 0.0001).

To observe the potential cardiotoxicity of fenpropathrin, the zebrafish was incubated at sub-lethal concentrations of 0.01, 0.1, and 1 ppm for 24 h. After incubation at 1 ppm of fenpropathrin, an observably bigger ventricle size was noted when compared with the control group, which is a sign of cardiomegaly (Figure 2A,B). A significant increase in the stroke volume, cardiac output, ejection fraction, and shortening fraction of zebrafish larvae was observed after exposure to fenpropathrin, which provides additional qualitative evidence in support of the morphological alterations (Figure 2C–F). A significant increase in the cardiac physiology parameters was followed by some dose-dependent increase in the heart rate, which could be the reason for the significant increments in the cardiac output (Figure 2G). However, no significant change in heart rate variability was observed after exposure to fenpropathrin based on sd1 and sd2 values extracted from the Poincare plot, even at 1 ppm concentration (Figure 2H,I).

**Figure 3:**
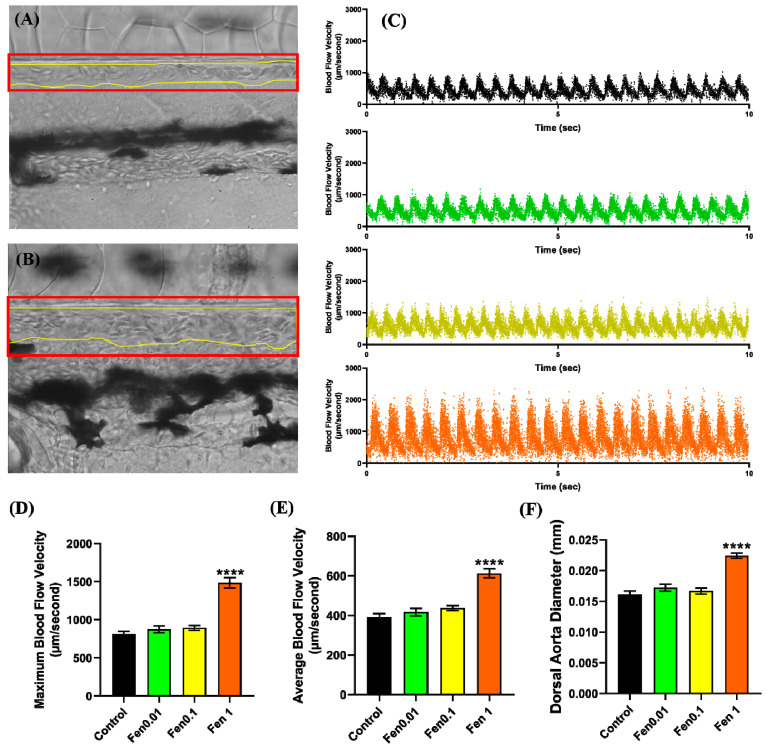
Condition of the dorsal aorta of control group (**A**) and the 1 ppm fenpropathrin-treated group (**B**). The yellow line represents the border of the dorsal aorta, while the red box represents its position for high-speed videography. (**C**) The blood flow velocity profile in the dorsal aorta following incubation in fenpropathrin concentrations of either zero (black), 0.01 (green), 0.1 (yellow), or 1 ppm (orange). (**D**) Maximum blood flow velocity, (**E**) average blood flow velocity, and (**F**) dorsal aorta diameter following 24 h of acute incubation in fenpropathrin. The data were presented as mean ± SEM, and the statistical significance was determined using Ordinary One-Way ANOVA with the Dunnet multiple comparison test. (**** *p* < 0.0001).

Based on the cardiac performance results, a follow-up assessment of the vascular system was performed by evaluating the vascular performance of zebrafish after fenpropathrin exposure. The results showed a significantly bigger vessel diameter in every fish observed in the 1 ppm fenpropathrin exposure group (Figure 3A,B,F). Changes in the blood flow profile are also observed in zebrafish exposed to 1 ppm of fenpropathrin, with considerably higher maximums and average velocity than the control group (Figure 3C–E). Based on these findings, we can conclude that fenpropathrin can elicit morphological and physiological changes in the zebrafish vascular system, as evidenced by a dilated dorsal aorta and increased blood flow velocity.

**Figure 4:**
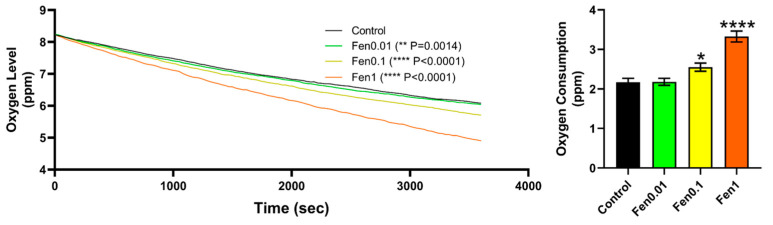
Oxygen consumption rate of zebrafish after exposure to fenpropathrin. The left graph depicts the oxygen level over time, showing a clear increase in oxygen consumption. The right graph shows the total oxygen consumption of zebrafish at the end of the test, which was significantly higher for fenpropathrin-exposed group compared to control group. Two-Way ANOVA was conducted to calculate the significant difference in the oxygen level over time between each treatment, and Geisser–Greenhouse correction was performed for multiple comparison tests. The data for total oxygen consumption were presented as mean ± SEM, and the statistical significance was calculated using Ordinary One-Way ANOVA with Dunnet multiple comparison test. (*n* = 46, * *p* < 0.05, **** *p* < 0.0001).

Another follow-up study was conducted to assess the metabolic rate of zebrafish after fenpropathrin exposure due to an elevation in some cardiovascular performance indices. The study evaluated the oxygen consumption of zebrafish following fenpropathrin exposure, and it was observed that there was a similar phenomenon with the cardiovascular performance parameters, whereby there was a significant increase in a dose-dependent manner beginning at 0.1 ppm (Figure 4). The data acquired from the study suggest that fenpropathrin can significantly raise cardiovascular parameters and metabolic rate, which could be linked to ventricle enlargement and some increases in heart rate.

### 3.2. Testing Fenpropathrin and Target Protein Interactions by Molecular Docking

**Figure 5:**
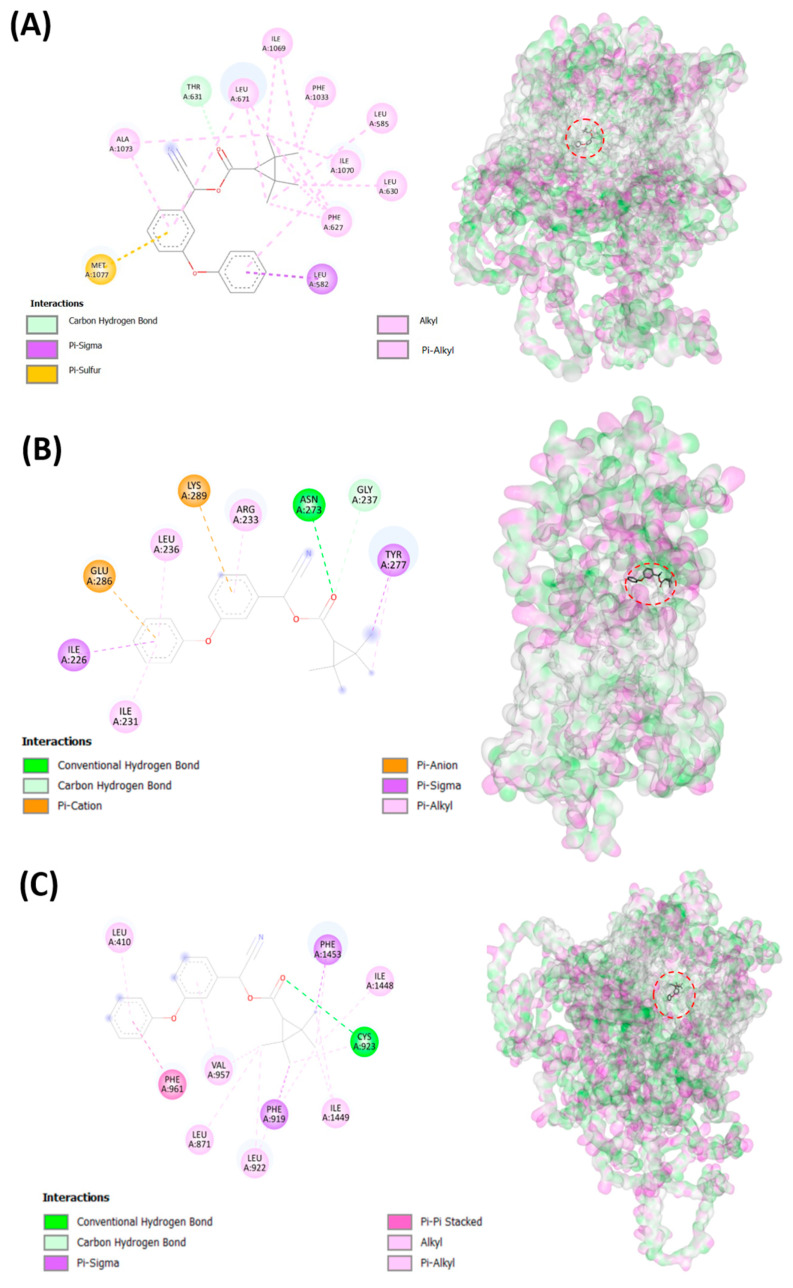

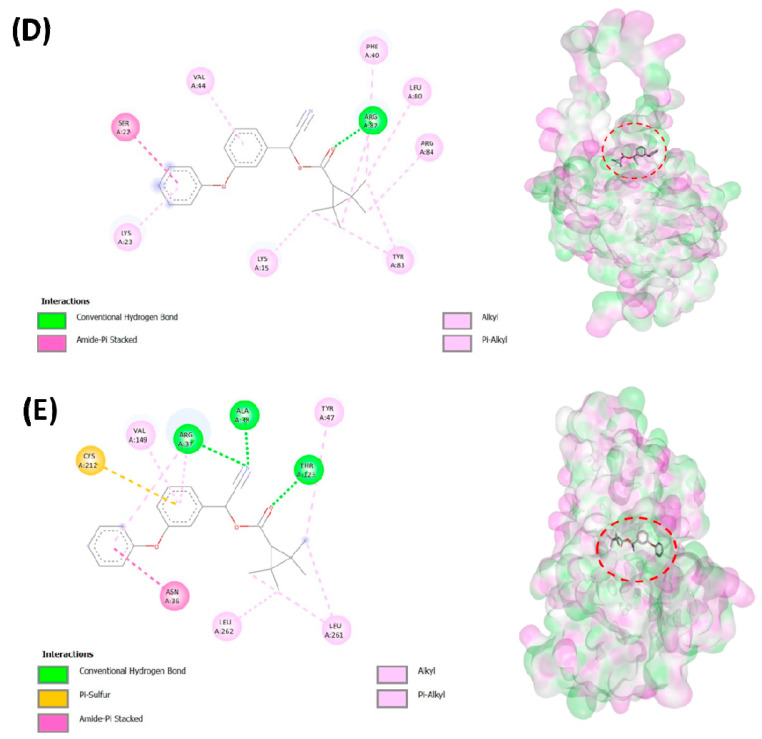
Graphical representation of the binding position of fenpropathrin into voltage-gated calcium (cacna1sb (**A**)), chloride (clcn3 (**B**)), and sodium channel (scn1lab (**C**)) and apoptosis-related gene bcl2a (**D**) and p53 (**E**). The dashed red line shows the position of fenpropathrin.

To elucidate the potential mechanism to induce cardiovascular alterations after fenpropathrin exposure, we conducted an in silico experiment by docking fenpropathrin with many different potential targets that have been reported in previous research. An in silico experiment using molecular docking was performed using several voltage-gated ion channels that have been previously reported to become disrupted after fenpropathrin exposure [36,37,38]. The docking result shows that fenpropathrin virtually binds with several voltage-gated ion channels with relatively high affinity. From the data, it can be observed that fenpropathrin obtained the highest binding energy, with scn1lab having a score of −9.7 kcal/mol (Table 3). The compound interacts with scn1lab through a conventional Hydrogen bond with Cys923; a Pi-Alkyl bond with Leu410 and Val957; an Alkyl bond with Leu871, Leu922, Ile1448, and Ile1449; a Pi-Sigma bond with Phe919 and Phe1453; and a Pi-Pi Stacked with Phe961. Interestingly, fenpropathrin also shows good binding energy with chloride channel, clcn3, exhibiting a conventional Hydrogen bond with Asn273; a Carbon Hydrogen bond with Gly237; Pi-Sigma with Ile226 and Tyr277; Pi-Anion with Glu286; Pi-Cation with Lys28; and Pi-Alkyl bonds with Ile231, Arg233, and Leu236 (Figure 5B). Furthermore, it also interacts with the calcium channel, cacna1sb, with good affinity via Carbon Hydrogen Bond with Thr63, Pi-Sigma with Leu582, Pi-Sulfur with Met1077, Alkyl with Leu585, Leu630, Leu671 (2), Ile1069 (2), Ile1070, Ala1073, and Pi-Alkyl with Phe627 (4), Leu671, Phe1033, Ile1070, and Ala1073. The visualization of 2D bonds and 3D docking of the fenpropathrin binding site to the mentioned proteins can be observed in Figure 5, while the binding position with other proteins can be observed in Table S1. All of those channels are expressed in cardiac muscle and function to maintain the balance of the cell membrane’s potential, so fenpropathrin cardiotoxicity might come from the blockage of those voltage-gated ion channels. Afterward, fenpropathrin’s binding affinity to several apoptosis-related proteins was also checked, as previous studies suggest that fenpropathrin causes the generation of reactive oxygen species (ROS) [12]. This was performed by checking the affinity with bcl2a, p53, and mdm2, and fenpropathrin showed a good binding affinity with bcl2a and p53. With bcl2a, fenpropathrin bound via the conventional Hydrogen Bond with Arg87, Amide-Pi Stacked with Ser22, Alkyl with Lys15, Leu80, Arg84, and Arg87, and Pi-Alkyl with Lys23, Phe40, Val44, and Tyr83 (2), while with p53, it bound via the conventional Hydrogen Bond with Arg37, Ala38, and Thr123, Pi-Sulfur with Cys212, Amide-Pi Stacked with Asn36, and Alkyl with Tyr47, Leu261 (2), and Leu262, and Pi-Alkyl with Arg37 (2) and Val149 (Figure 5).

### 3.3. Testing Marker Gene Expression after Fenpropathrin Exposure

**Figure 6:**
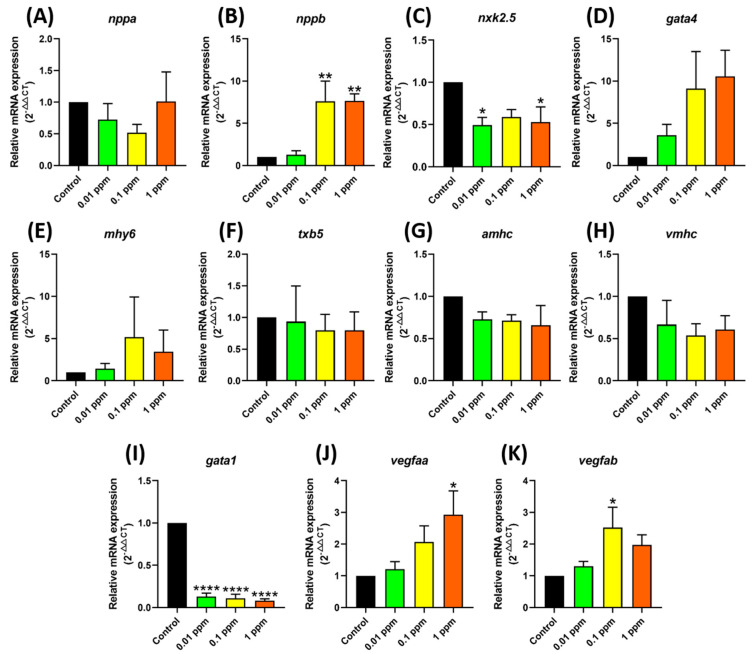
Expression of genes related to cardiovascular development after exposure to fenpropathrin. The data were presented as mean ± SEM, and the statistical significance was calculated using Ordinary One-Way ANOVA with Dunnet multiple comparison test. (*n* = 4, * *p* < 0.05, ** *p* < 0.01, **** *p* < 0.0001).

As part of our investigation into the toxicity caused by fenpropathrin, we conducted a detailed analysis with qRT-PCR to evaluate some important marker gene expressions at the mRNA level. The results revealed significant changes in several genes related to cardiovascular development, particularly at the highest concentration tested. Notably, significant upregulation of the cardiac damage marker of *nppb* after incubation with 0.1 and 1 ppm (Figure 6B) fenpropathrin was observed, which was consistent with the cardiomegaly phenotype detected in previous tests. Additionally, the expression of *vegfaa* and *vegfab* was upregulated at 0.1 and 1 ppm, respectively, as evidenced by a significant change in the cardiovascular performance parameters (Figure 6J,K). On the other hand, a sharp decline of *gata1* after fenpropathrin exposure was observed, indicating that fenpropathrin might cause damage to zebrafish red blood cells (Figure 6I).

## 4. Discussion

This study provides the first concerning finding that cardiomegaly might occur in zebrafish after acute fenpropathrin exposure. Cardiomegaly, or an enlarged heart, is one phenotype that is usually caused by multiple cardiovascular-related problems. Cardiomegaly can result in cardiac dilatation or hypertrophy, leading to clinical heart failure syndrome. There are several known causes of cardiomegaly, including coronary artery disease, either by myocardial infarction or ischemia; hypertensive heart disease; congenital heart disorder; pulmonary diseases; arrhythmia; systemic diseases that lead to a high output state; and even lung cancer [39,40,41,42]. However, very few studies have explored the impact of fenpropathrin exposure on cardiovascular physiology. Previous studies have shown that other synthetic pyrethroids like ectomethrin can cause myocardial hypertrophy with elongated hyperchromatic nuclei in rat hearts [43]. Another study showed that α-cypermethrin, type II pyrethroids, can cause hypertrophic cardiomyocytes and myocardial fibrosis in rats [44]. These findings suggest the possibility of cardiotoxicity caused by pyrethroids.

Aside from cardiomegaly, significant heart rate and blood flow velocity increments were observed in zebrafish, along with vasodilation in the dorsal aorta. Vasodilation refers to the widening of a blood vessel to allow more blood to flow through and lower the blood pressure [45]. It happens naturally in the body to enhance blood flow to specific areas of the body, but excessive vasodilation could lead to anaphylaxis and septic shock [46,47]. Vasodilation happens when the smooth muscle surrounding the blood vessel relaxes, and one of the major contributors to this process is the homeostasis of the intracellular calcium ion [48,49]. Several side effects of vasodilation after pyrethroid exposure have been reported, although they are not specific to arterial vasodilation. Fernandes et al. (2020) reported vasodilation in the gills after λ-cyhalothrin exposure in tilapia [50]. Hemodynamic instability also occurred in some patients who were poisoned with λ-cyhalothrin [51]. It has been established that pyrethroids are potent releasers of neurotransmitters, including dopamine, and they affect the calcium balance inside the cells [52,53,54,55,56]. Dopamine is an intriguing compound that functions as a vasodilator at low concentrations, but at high concentrations, it works as a vasoconstrictor [54]. However, fenpropathrin is an exception to this rule among pyrethroids because it induces dopaminergic degeneration, which eliminates the possibility of vasodilation caused by the dopaminergic system [12,14].

It is well known that the toxicity of pyrethroids occurs through the activation of a voltage-gated sodium channel. Based on the research by Bradberry et al. (2005), it has been suggested that pyrethroids can be highly toxic to insects due to their increased sensitivity to sodium channels [26]. This is because pyrethroids can modify the gating characteristic of sodium channels, which can cause a delay in their closure and lower the action potential threshold. As a result, repetitive firing can occur if the delay is prolonged enough [26,57,58].

It is interesting to note that pyrethroids, which were previously known for their effect on voltage-gated sodium channels, also affect other ion channels such as calcium and chloride channels [3,59]. In fact, fenpropathrin has a high binding affinity to every voltage-gated channel tested, according to the molecular docking data. Studies have established the effect of pyrethroids on voltage-gated calcium channels [60,61,62]. Fenpropathrin has been reported to increase the concentration of intracellular Ca^2+^ after its action on voltage-gated sodium channels in neocortical neurons [37]. However, an extended increase in intracellular Ca^2+^ can be harmful to the cell as it can lead to the accumulation of ROS, mitochondrial dysfunctions, and neuronal death [16,63]. In this study, aside from an increase in the ventricle size, a significant increase in blood flow velocity and oxygen consumption was observed after fenpropathrin exposure. The Ca^2+^ concentration during systole determines the contractility of the heart. This can be attributed to the possibility of an influx of Ca^2+^ caused by fenpropathrin, which leads to stronger contractility of the heart and increased energy [64,65,66].

It has been found that the chloride channel plays a crucial role in regulating the basal membrane potential and affecting the swelling and stretching of cardiac myocytes. This channel also regulates the cardiac electrical activity of the heart, myocyte volume, apoptosis, and ischemic preconditioning [67,68,69]. Cell size regulation is carried out through a swelling-activated chloride current (I_CL,sweel_) [68]. The involvement of pyrethroids in chloride channels was first discovered through experiments using deltamethrin and cismethrin for chloride conductance in rat muscle and voltage-gated chloride channels in neuroblastoma cells [70]. These experiments show that rats treated with ivermectin and pentobarbital, which are known as voltage-gated chloride channel activators, showed a reduced degree of salivation and a better motor response after intravenous treatment of deltamethrin, a type II pyrethroid [71]. Another study utilized the patch clamp technique on N1E-115 neuroblastoma, showing that deltamethrin blocks the voltage-gated chloride channel conductance [72]. Molecular docking data show a high affinity of fenpropathrin to every kind of chloride channel tested, leading to the hypothesis that the blockage of those chloride channels could result in osmotic instability of intracellular ions and cause the cells to swell to maintain the ion balance; this could potentially lead to cardiomegaly.

Gene expression analysis shows that the expression of *gata1* was significantly downregulated in the fenpropathrin-exposed group. The GATA1 protein is considered one of the key proteins in erythropoiesis [73,74]. Up until this manuscript’s making, no further study had focused on the effect of pyrethroids on hematopoiesis. However, Luty et al. (2001) reported that deltamethrin and fenvalverate disturbed hemoglobin synthesis, resulting in significantly lower erythrocytes in female Swiss mice [75]. Khan et al. (2009) also reported that cypermehtrin could disturb the red blood cell and heme biosynthesis in dwarf goats [76]. Hematological alteration was also observed in water buffalo calves that were dermally exposed to cypermethrin [77]. Another recent study performed by Guo et al. (2023) also showed that the level of *gata1a* was significantly downregulated when zebrafish were exposed with tralomethrin [78]. Based on those previous studies, it is safe to say that pyrethroids could alter hematogenesis by altering the *gata1* gene expression, although more studies need to be performed to further validate this issue.

**Figure 7:**
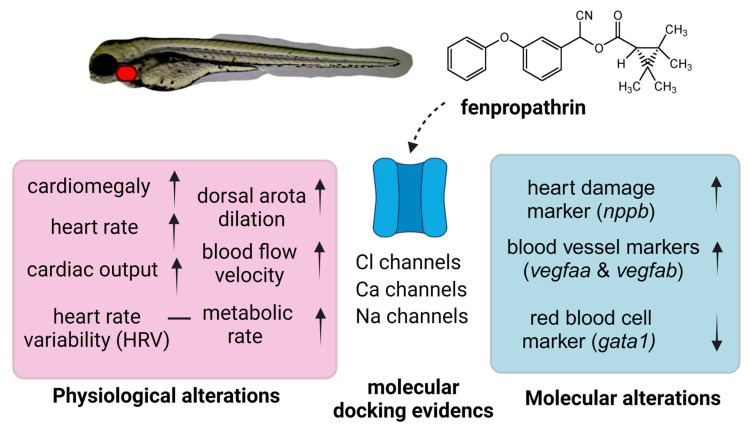
Proposed mechanism of fenpropathrin-induced cardiovascular toxicity in zebrafish. The physiological evidence was collected from cardiovascular and metabolic assays and is highlighted in red. The molecular evidence was collected from mRNA expression assay and is highlighted in blue. Some indirect evidence collected from molecular docking is highlighted with dotted arrow.

The current study revealed that fenpropathrin could bind with several voltage-gated ion channels and apoptotic-related proteins with high binding energy. Leading to our hypothesis that the toxicity of fenpropathrin is related to the alteration in the function of multiple ion channel together at once, along with an imbalance of ROS production. It is worth mentioning that fenpropathrin is a unique pyrethroids that is categorized into both type I and type II pyrethroids due to its poisoning syndrome [79]. However, this hypothesis needs further validation using multiple drugs related to the corresponding ion channel and antioxidants that can help alleviate the ROS damage. The proposed mechanism of fenpropathrin toxicity is summarized in Figure 7.

## 5. Conclusions

The current study has revealed that fenpropathrin causes cardiotoxicity in zebrafish larvae. The elevated heart size, tachycardia, vasodilation, high blood flow velocity, and higher metabolic rate seen in zebrafish after exposure to this substance suggest that fenpropathrin negatively affects the cardiovascular system. This finding was further supported by qRT-PCR data, which showed significant alterations in several cardiovascular-related markers following fenpropathrin exposure. Molecular docking data revealed that fenpropathrin exhibited a high affinity or voltage-gated sodium, chloride, and calcium channels and apoptosis-related proteins p53 and bcl2a that might disturb its function, causing a significant increase in ROS, as reported in a previous study. This study highlights the need for further research into fenpropathrin cardiotoxicity, as little research has been conducted on this issue.

## Figures and Tables

**Table 1:** List of the primers used in the study.

Gene	Forward Primer (5′-3′)	Reversed Primer (5′-3′)
*myh6*	F: CACCAGCAGACACTGGATG	R: GCTCCAAGTCCATTCTGAC
*tbx5*	F: ATTCGCCGATAACAAATGG	R: CGCCTTGACGATGTGGAT
*vmhc*	F: GAAGAGGCAGAGGCATCACT	R: AATTGCGTTTGCTCTGCTCC
*amhc*	F: AAGCCACTACCGCCTCTCTA	R: TTTGAGGCAAGGTCGTCCAA
*nxk2.5*	F: GTCCAGGCAACTCGAACTACTC	R: AACATCCCAGCCAAACCATA
*gata4*	F: TCCAGGCGGGTGGGTTTATC	R: TGTCTGGTTCAGTCTTGATGGGTC
*vegfaa*	F: AAAAGAGTGCGTGCAAGACC	R: GACGTTTCGTGTCTCTGTCG
*vegfab*	F: TGTTGGTGGAAATTCAGCAG	R: CACCCTGATGACGAAGAGGT
*nppa*	F: AAGCAAAAGCTTGTCTGG	R: ACTGTATCCGCATATTGCAGC
*nppb*	F: CATTCCCGTAGTCGGCCTTC	R: CTTCAATATTTGCCGCCTTTAC

**Table 2:** Grid Box Parameters.

Gene	Protein	UniProt	Coordinates and Dimension of the Grid Box
*cacna1fa*	Voltage-dependent L-type calcium channel subunit alpha	F1R736	Center: x = −21.175, y = −1.963, z = 8.991 Dimensions (Å): x = 83.040, y = 64.471, z = 92.663
*cacna1g*	Voltage-dependent T-type calcium channel subunit alpha	e7f9f6	Center: x = −22.230, y = −6.492, z = 22.740 Dimensions (Å): x = 116.140, y = 81.918, z = 124.875
*cacna1ha*	Calcium channel, voltage-dependent, T type, alpha 1H subunit a	A0A2R8PWF8	Center: x = 3.168, y = 6.847, z = 12.372 Dimensions (Å): x = 86.560, y = 63.668, z = 60.626
*cacna1sb*	Voltage-dependent L-type calcium channel subunit alpha	Q6RKB0	Center: x = −23.067, y = −10.319, z = 10.135 Dimensions (Å): x = 106.084, y = 103.402, z = 72.540
*clcn1a*	Chloride channel, voltage-sensitive 1a	A0A0R4ID92	Center: x = 2.964, y = 8.362, z = 7.264 Dimensions (Å): x = 34.792, y = 33.053, z = 29.360
*clcn1b*	Chloride channel, voltage-sensitive 1b	A0A2R8Q7B5	Center: x = 1.488, y = −0.489, z = −0.657 Dimensions (Å): x = 44.666, y = 30.449, z = 36.670
*clcn2a*	Chloride channel, voltage-sensitive 2a	A5PMN6	Center: x = 0.671, y = 3.518, z = 15.953 Dimensions (Å): x = 66.344, y = 77.634, z = 108.854
*clcn2b*	Chloride channel protein	A0A098DN12	Center: x = −3.741, y = 6.447, z = −1.055 Dimensions (Å): x = 44.981, y = 31.420, z = 39.037
*clcn3*	Chloride channel protein	A0A140LGW7	Center: x = 2.777, y = 1.185, z = 5.672 Dimensions (Å): x = 61.361, y = 42.659, z = 56.203
*scn1lab*	Sodium channel protein	F1QXF5	Center: x = −20.241, y = 2.816, z = −1.385 Dimensions (Å): x = 45.601, y = 38.333, z = 44.803
*scn4aa*	Voltage-gated sodium channel type 4 subunit alpha A	Q2XVR3	Center: x = −22.588, y = 3.698, z = 1.059 Dimensions (Å): x = 55.172, y = 49.391, z = 71.775
*scn4ab*	Sodium channel protein type 4 subunit alpha B	Q20JQ7	Center: x = −2.948, y = 8.484, z = 7.078 Dimensions (Å): x = 86.715, y = 130.031, z = 121.072
*scn5lab*	Voltage-gated sodium channel type V-like, alpha B	A0A0G2KYI1	Center: x = −24.519, y = 1.712, z = 18.384 Dimensions (Å): x = 64.739, y = 50.480, z = 71.871
*scn8aa*	Sodium channel 8	A0A2R8QLY1	Center: x = −25.787, y = 12.332, z = −9.831 Dimensions (Å): x = 56.054, y = 47.937, z = 47.219
*bcl2a*	Apoptosis regulator Bcl-2	Q564A4	Center: x = −0.361, y = 1.914, z = 5.890 Dimensions (Å): x = 36.912, y = 45.937, z = 37.158
*mdm2*	E3 ubiquitin-protein ligase Mdm2	Q561Z0	Center: x = −1.626, y = 2.424, z = 12.751 Dimensions (Å): x = 45.601, y = 38.333, z = 44.803
*p53*	Tumor protein p53 inducible protein 3	A0A286YBA3	Center: x = −20.241, y = 2.816, z = −1.385 Dimensions (Å): x = 45.601, y = 38.333, z = 44.803

**Table 3:** Binding energy of fenpropathrin with voltage-gated sodium channels of zebrafish.

Gene	Binding Energy (kcal/mol)	Gene	Binding Energy (kcal/mol)
*cacna1fa*	−8.3	*scn1lab*	−9.7
*cacna1g*	−7.4	*scn4aa*	−9.5
*cacna1ha*	−7.5	*scn4ab*	−9.4
*cacna1sb*	−8.6	*scn5lab*	−9.5
*clcn1a*	−8.2	*scn8aa*	−9.5
*clcn1b*	−8.1	*bcl2a*	−8.5
*clcn2a*	−8.6	*p53*	−8.4
*clcn2b*	−8.5	*mdm2*	−6.4
*clcn3*	−8.7		

## Data Availability

We have full control of all primary data and agree to allow the journal to review our data upon request.

## Supplementary Materials

The following supporting information can be downloaded at: https://www.mdpi.com/article/10.3390/biology12091214/s1, Figure S1. Cardiac performance parameter after acute 24 h incubation in chlorantriniprolle at different concentration from 0.01 to 10 ppm (A–G). The data was presented as mean ± statistical error mean (SEM), and the statistical significance was calculated using Ordinary One-Way ANOVA with Dunnet multiple comparison test. (* *p* < 0.05, ** *p* < 0.01). Figure S2. Cardiac performance parameter after acute 24 h incubation in diuron chlorantriniprolle at different concentration from 0.01 to 10 ppm (A–G). The data was presented as mean ± statistical error mean (SEM), and the statistical significance was calculated using Ordinary One-Way ANOVA with Dunnet multiple comparison test. (* *p* < 0.05). Figure S3. Cardiac performance parameter after acute 24 h incubation in fipronil chlorantriniprolle at different concentration from 0.01 to 10 ppm (A–G). The data was presented as mean ± statistical error mean (SEM), and the statistical significance was calculated using Ordinary One-Way ANOVA with Dunnet multiple comparison test. (* *p* < 0.05). Figure S4. Cardiac performance parameter after acute 24 h incubation in imidacloprid chlorantriniprolle at different concentration from 0.01 to 10 ppm (A–G). The data was presented as mean ± statistical error mean (SEM), and the statistical significance was calculated using Ordinary One-Way ANOVA with Dunnet multiple comparison test. (* *p* < 0.05, ** *p* < 0.01, **** *p* < 0.0001). Figure S5. Cardiac performance parameter after acute 24 h incubation in oxyfluorfen chlorantriniprolle at different concentration from 0.01 to 10 ppm (A–G). The data was presented as mean ± statistical error mean (SEM), and the statistical significance was calculated using Ordinary One-Way ANOVA with Dunnet multiple comparison test. Figure S6. Cardiac performance parameter after acute 24 h incubation in paraquat chlorantriniprolle at different concentration from 0.01 to 10 ppm (A–G). The data was presented as mean ± statistical error mean (SEM), and the statistical significance was calculated using Ordinary One-Way ANOVA with Dunnet multiple comparison test. (* *p* < 0.05, **** *p* < 0.0001). Figure S7. Cardiac performance parameter after acute 24 h incubation in thiobencarb chlorantriniprolle at different concentration from 0.01 to 10 ppm (A–G). The data was presented as mean ± statistical error mean (SEM), and the statistical significance was calculated using Ordinary One-Way ANOVA with Dunnet multiple comparison test. (* *p* < 0.05, ** *p* < 0.01, **** *p* < 0.0001). Table S1. 2D interaction diagram and 3D docking snapshot of Fenpropathrin with the target gene.
