# Supplementary material for: The Effect of the Pyrethroid Pesticide Fenpropathrin on the Cardiac Performance of Zebrafish and the Potential Mechanism of Toxicity
Source: Biology (Basel). 2023 Sep 6;12(9):1214. doi: 10.3390/biology12091214 (PMC10525504; doi:10.3390/biology12091214)
Supplement: Supplementary file 1 [file biology-12-01214-s001.zip › Supplementary Figure.pdf]

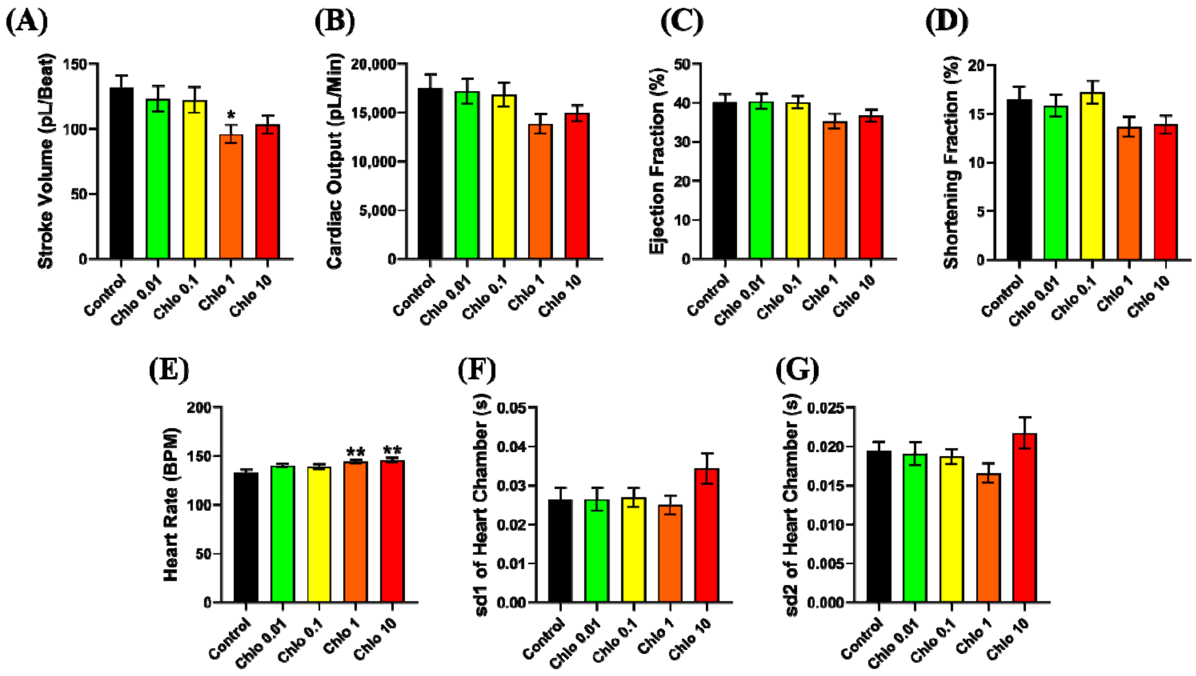

Figure S1. Cardiac performance parameter after acute 24 hours incubation in chlorantriniprolle at different concentration from 0.01 to 10 ppm (A-G). The data was presented as mean ± statistical error mean (SEM), and the statistical significance was calculated using Ordinary One-Way ANOVA with Dunnet multiple comparison test. (*p<0.05, **p<0.01).

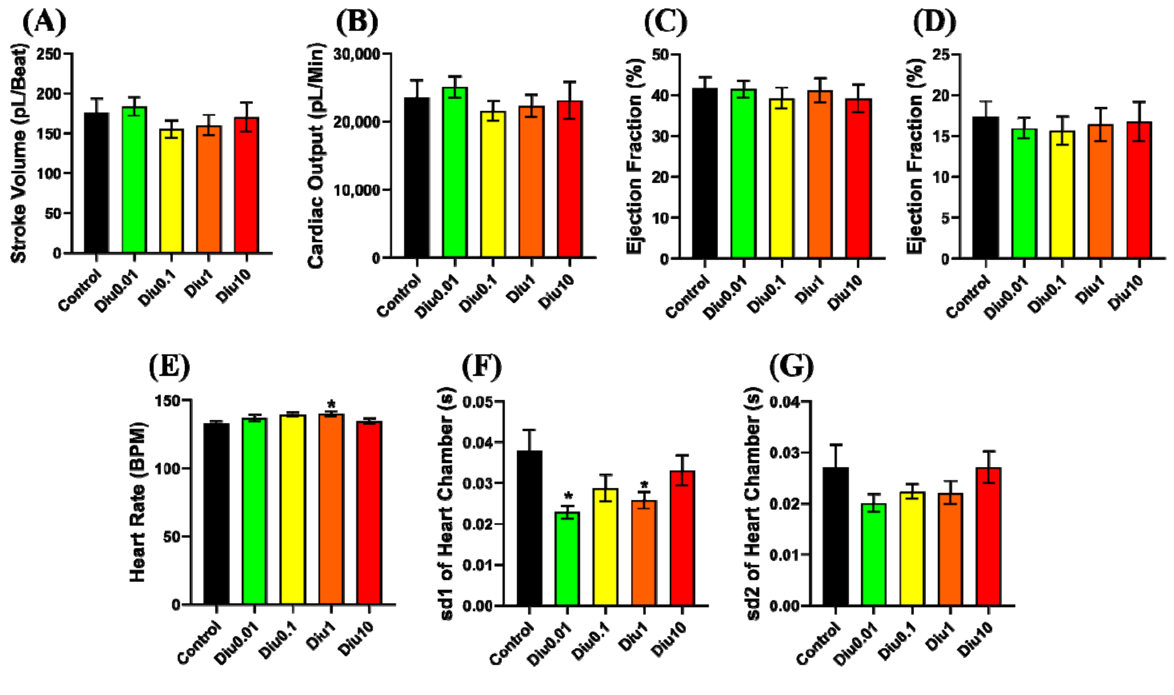

Figure S2. Cardiac performance parameter after acute 24 hours incubation in diuron chlorantriniprolle at different concentration from 0.01 to 10 ppm (A-G). The data was presented as mean ± statistical error mean (SEM), and the statistical significance was calculated using Ordinary One-Way ANOVA with Dunnet multiple comparison test. (*p<0.05).

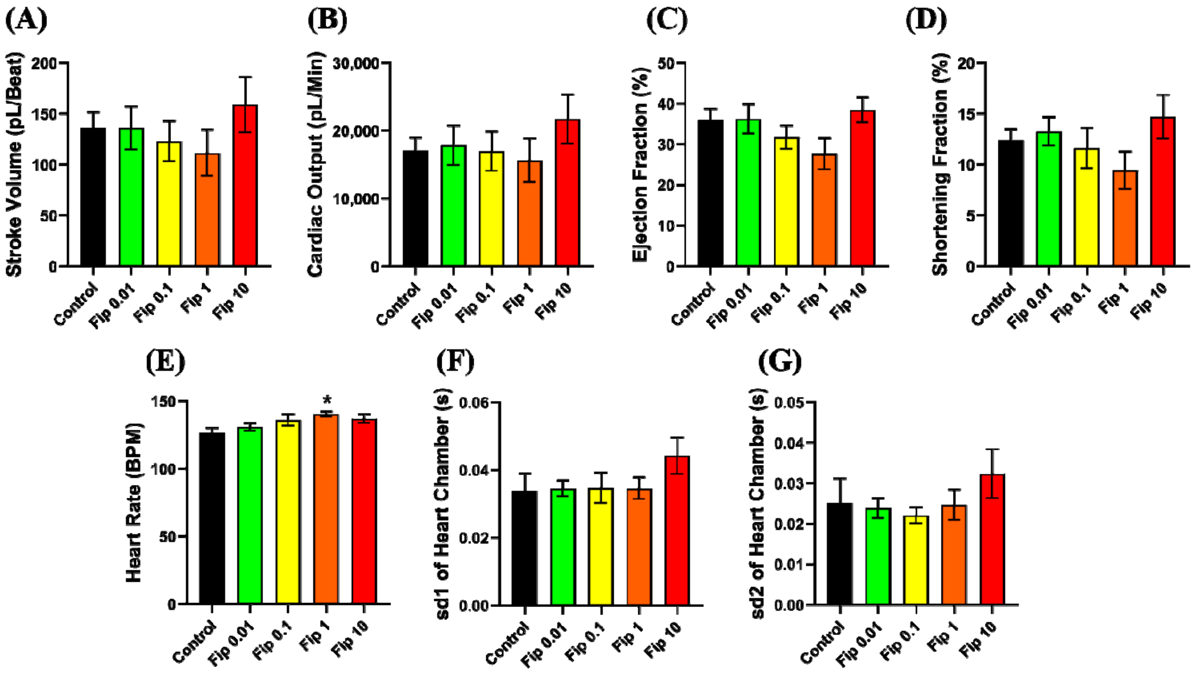

Figure S3. Cardiac performance parameter after acute 24 hours incubation in fipronil chlorantriniprolle at different concentration from 0.01 to 10 ppm (A-G). The data was presented as mean ± statistical error mean (SEM), and the statistical significance was calculated using Ordinary One-Way ANOVA with Dunnet multiple comparison test. (*p<0.05).

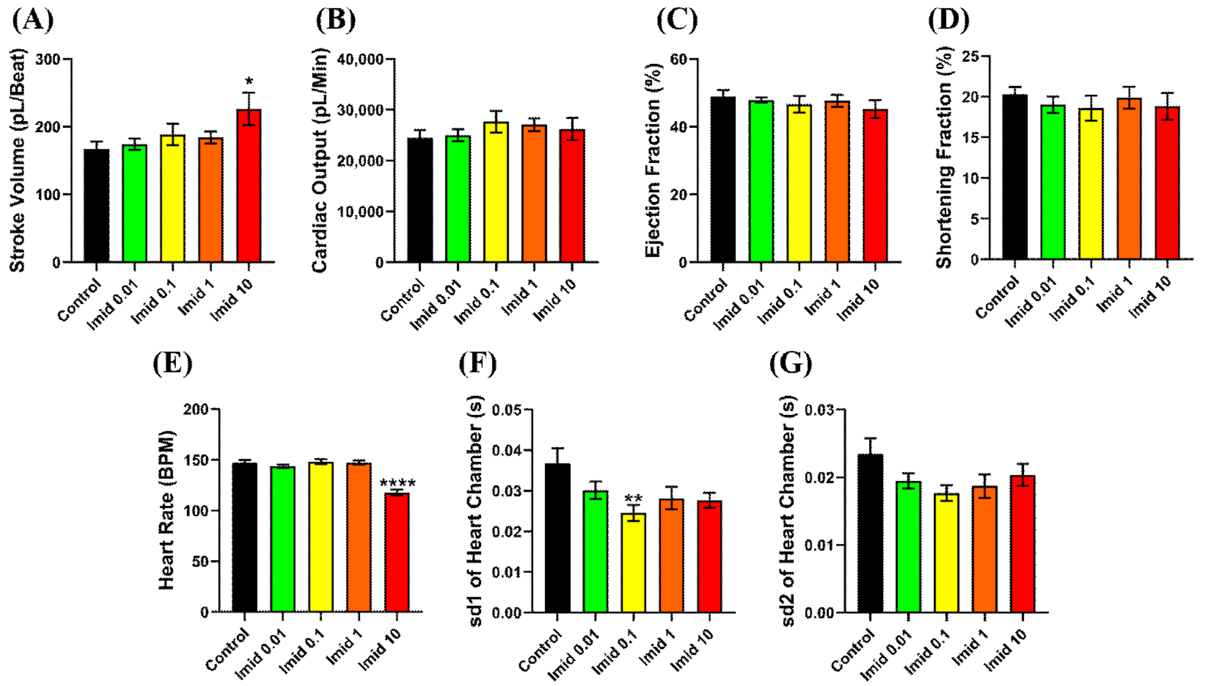

Fig. S4. Cardiac performance parameter after acute 24 hours incubation in imidacloprid chlorantriniprolle at different concentration from 0.01 to 10 ppm (A-G). The data was presented as mean ± statistical error mean (SEM), and the statistical significance was calculated using Ordinary One-Way ANOVA with Dunnet multiple comparison test. (*$p<0.05$, **$p<0.01$, ****$p<0.0001$).

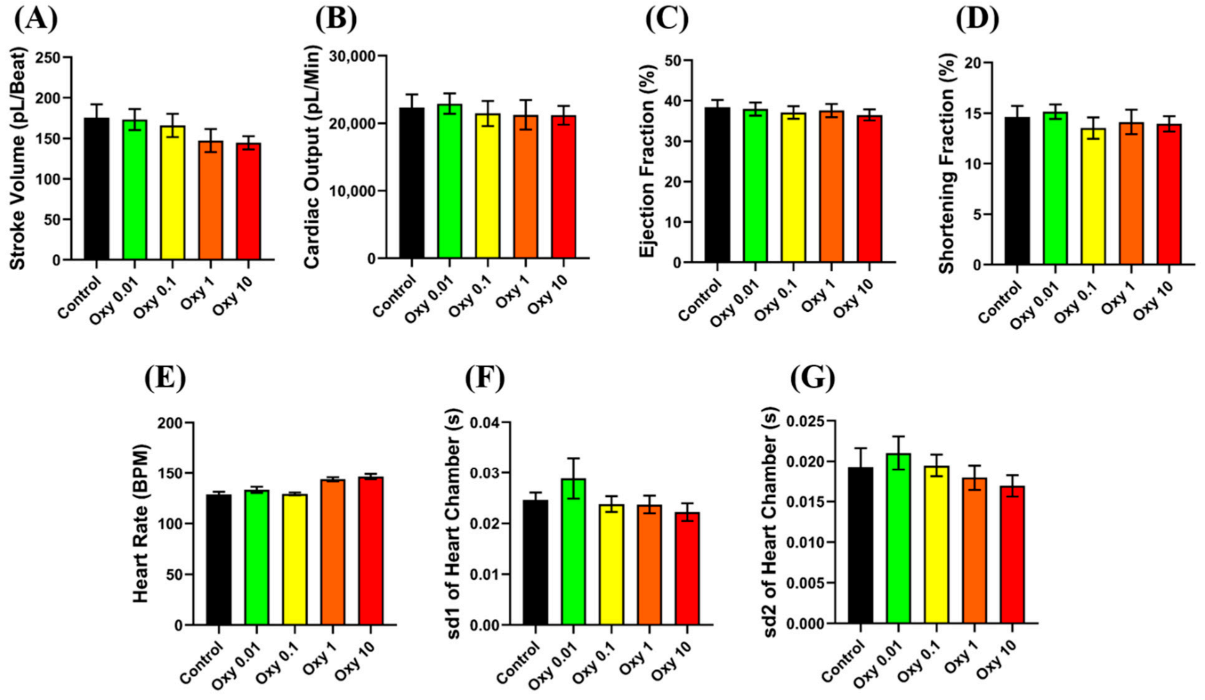

Fig. S5. Cardiac performance parameter after acute 24 hours incubation in oxyfluorfen chlorantriniprolle at different concentration from 0.01 to 10 ppm (A-G). The data was presented as mean ± statistical error mean (SEM), and the statistical significance was calculated using Ordinary One-Way ANOVA with Dunnet multiple comparison test.

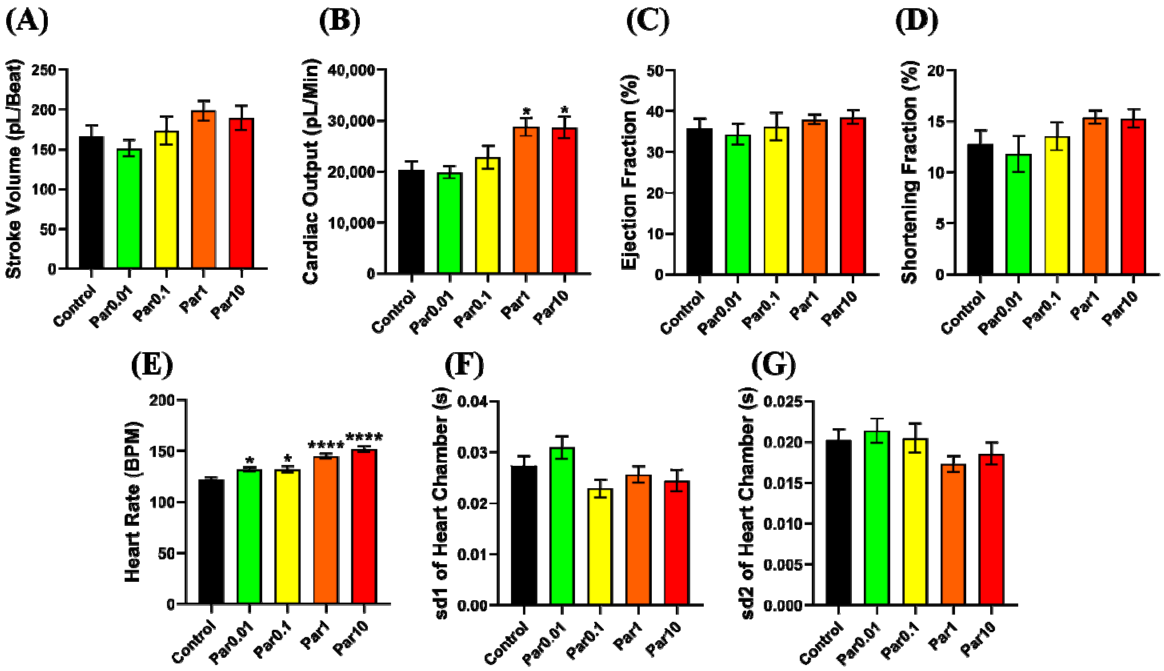

Fig. S6. Cardiac performance parameter after acute 24 hours incubation in paraquat chlorantriniprolle at different concentration from 0.01 to 10 ppm (A-G). The data was presented as mean ± statistical error mean (SEM), and the statistical significance was calculated using Ordinary One-Way ANOVA with Dunnet multiple comparison test. (*p<0.05, ****p<0.0001).

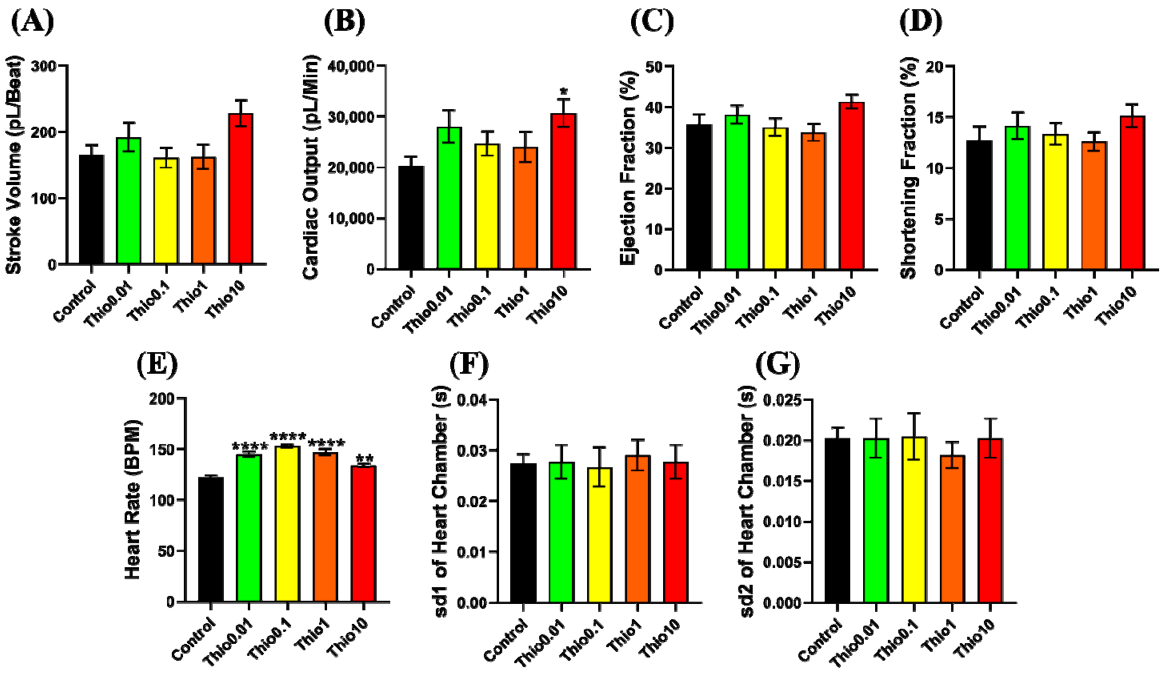

Fig. S7. Cardiac performance parameter after acute 24 hours incubation in thiobencarb chlorantriniprolle at different concentration from 0.01 to 10 ppm (A-G). The data was presented as mean ± statistical error mean (SEM), and the statistical significance was calculated using Ordinary One-Way ANOVA with Dunnet multiple comparison test. (*p<0.05, **p<0.01, ****p<0.0001).
