# Supplementary material for: The Effect of the Pyrethroid Pesticide Fenpropathrin on the Cardiac Performance of Zebrafish and the Potential Mechanism of Toxicity
Source: Biology (Basel). 2023 Sep 6;12(9):1214. doi: 10.3390/biology12091214 (PMC10525504; doi:10.3390/biology12091214)

Table S1. 2D interaction diagram and 3D docking snapshot of Fenpropathrin with the target gene

<table>
<tr><th>Gene</th><th>Key residues of interactions</th></tr>
<tr><td>scn5lab</td><td>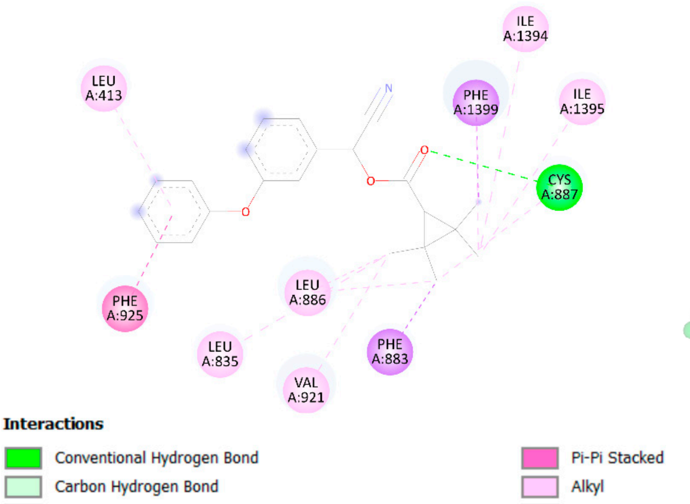

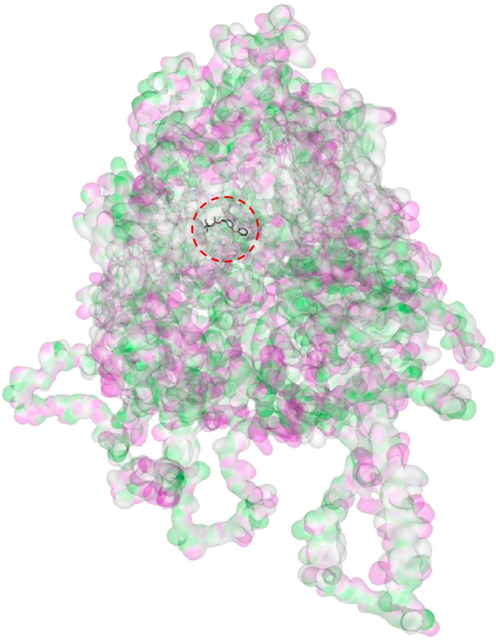</td></tr>
<tr><td>scn4aa</td><td>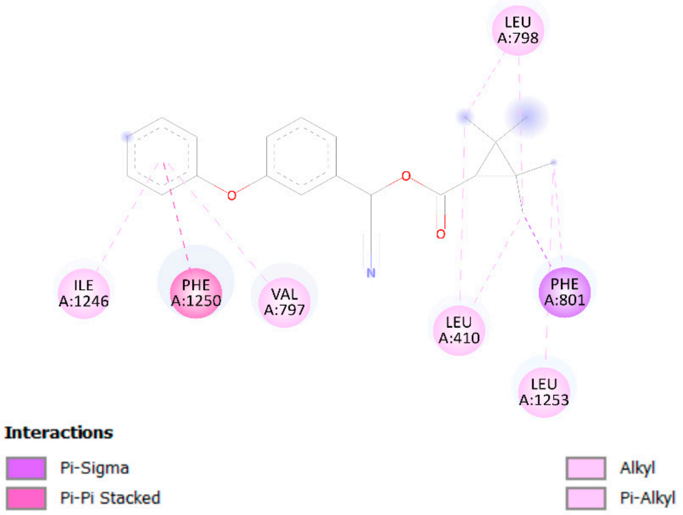

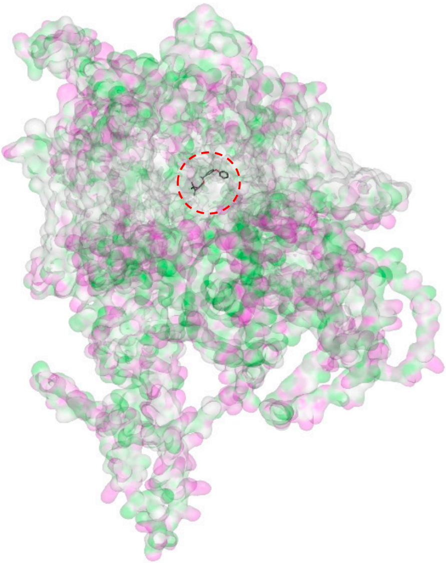</td></tr>
</table>

scn4ab	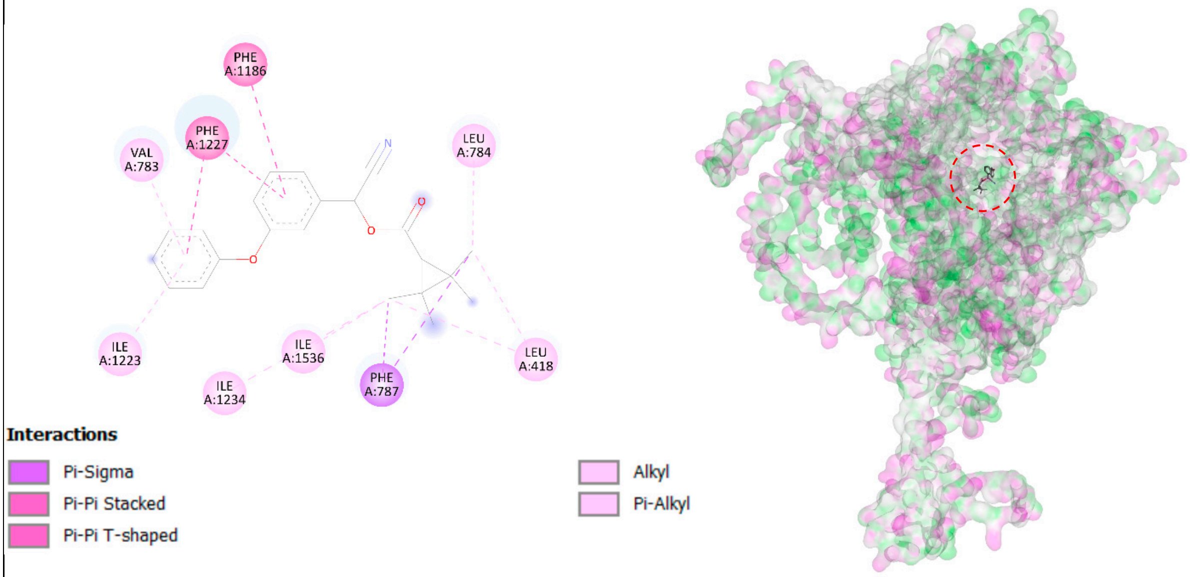
scn8aa	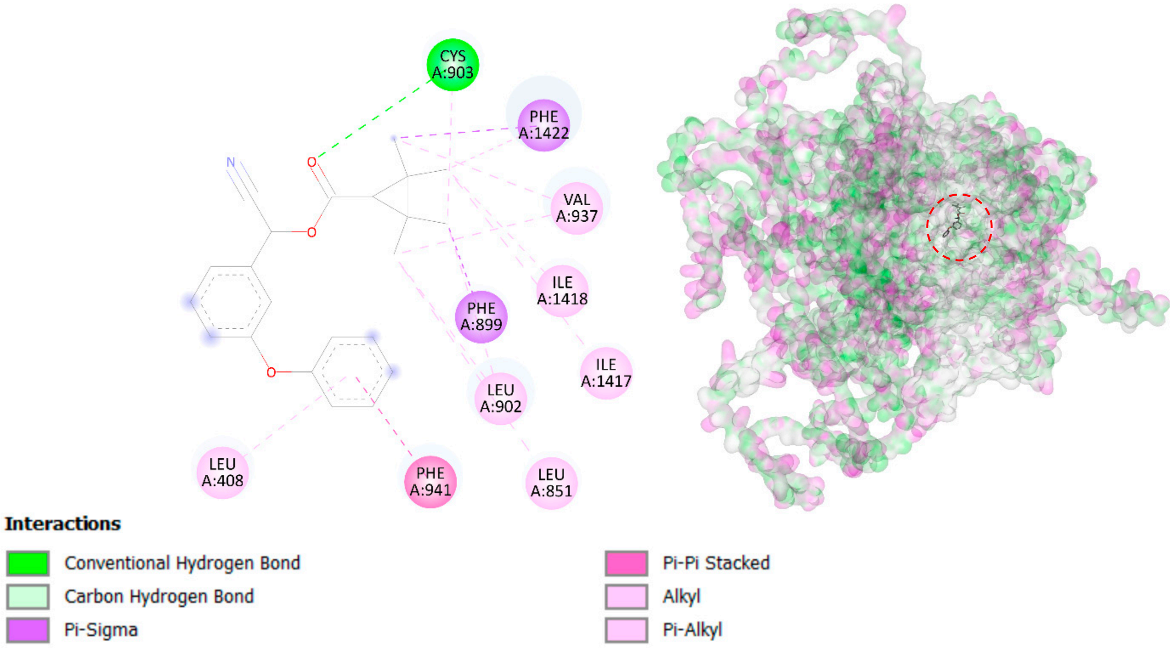

clcn1a	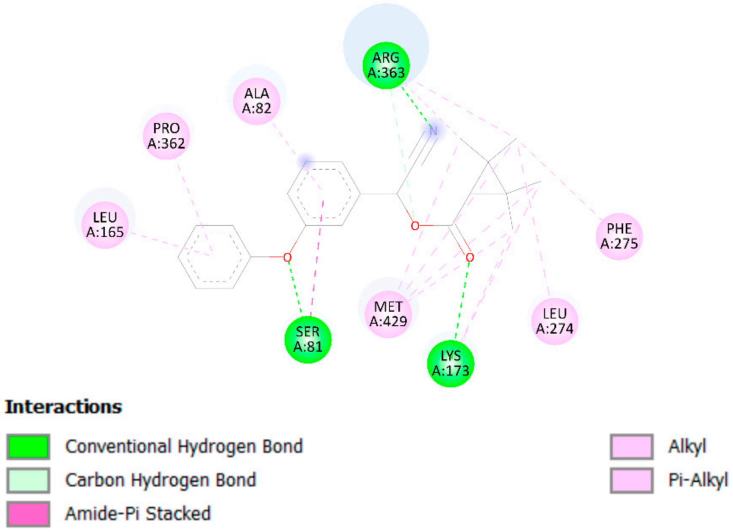 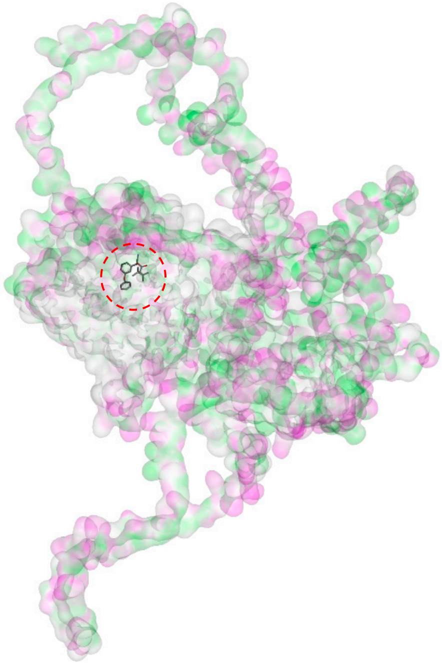
clcn1b	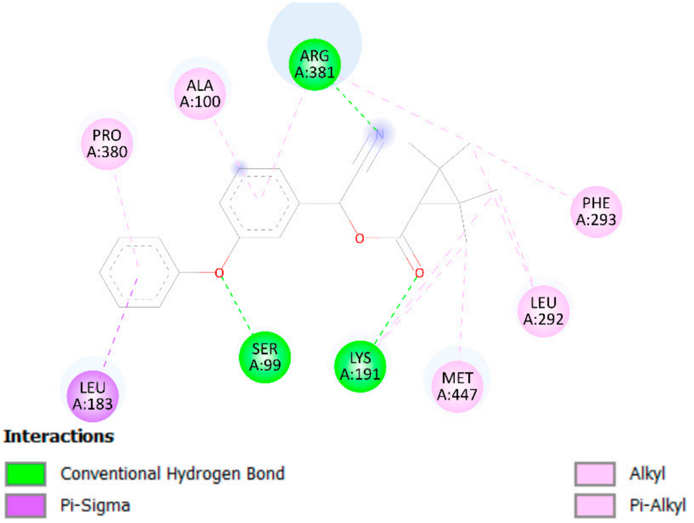 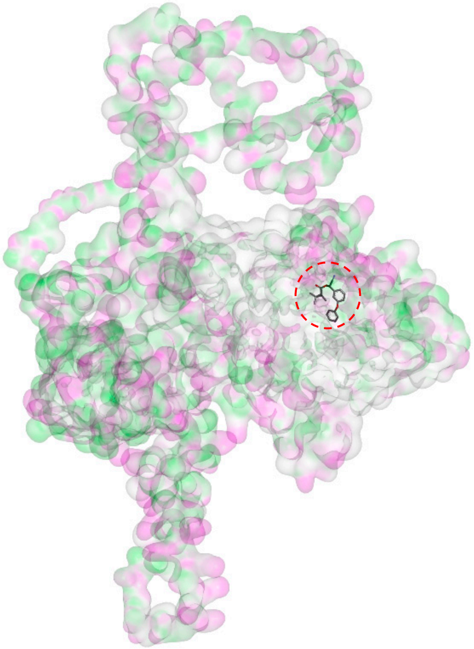

<table>
<tr>
<td>clcn2a</td>
<td>
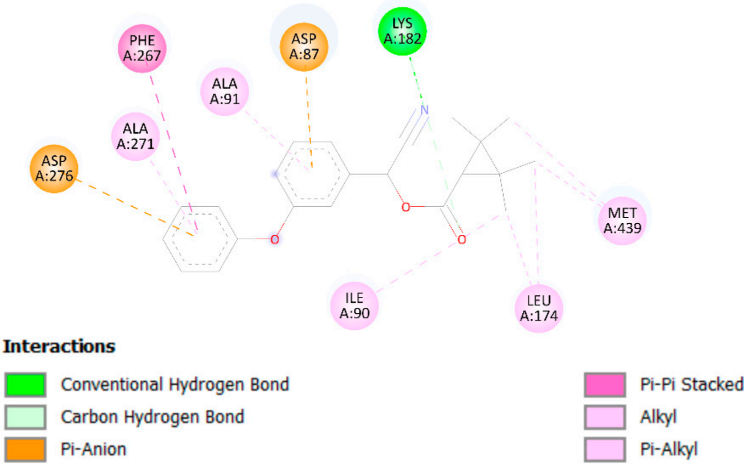

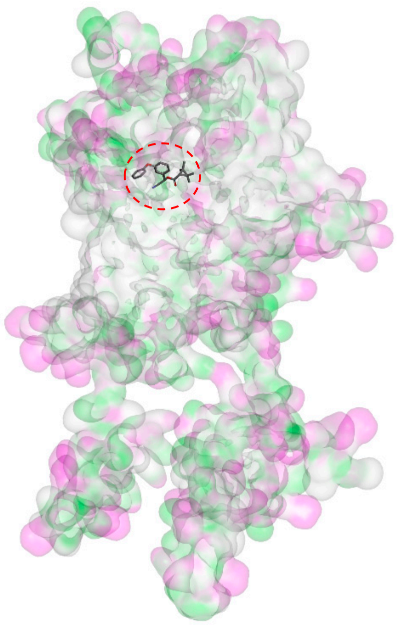
</td>
</tr>
<tr>
<td>clcn2b</td>
<td>
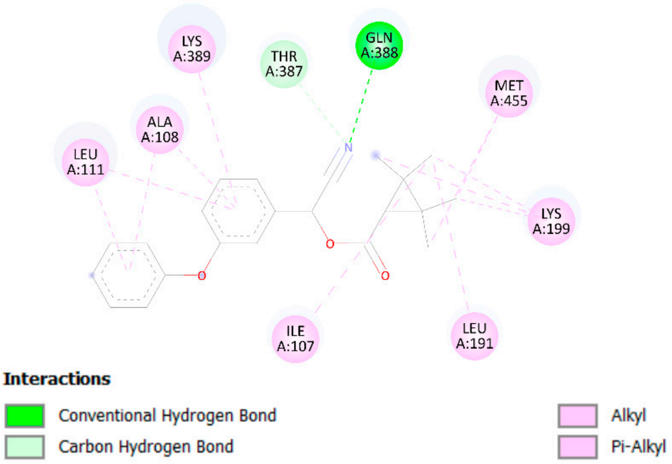

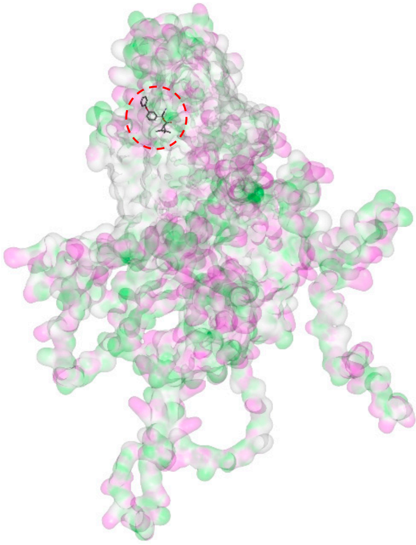
</td>
</tr>
</table>

cacna1g	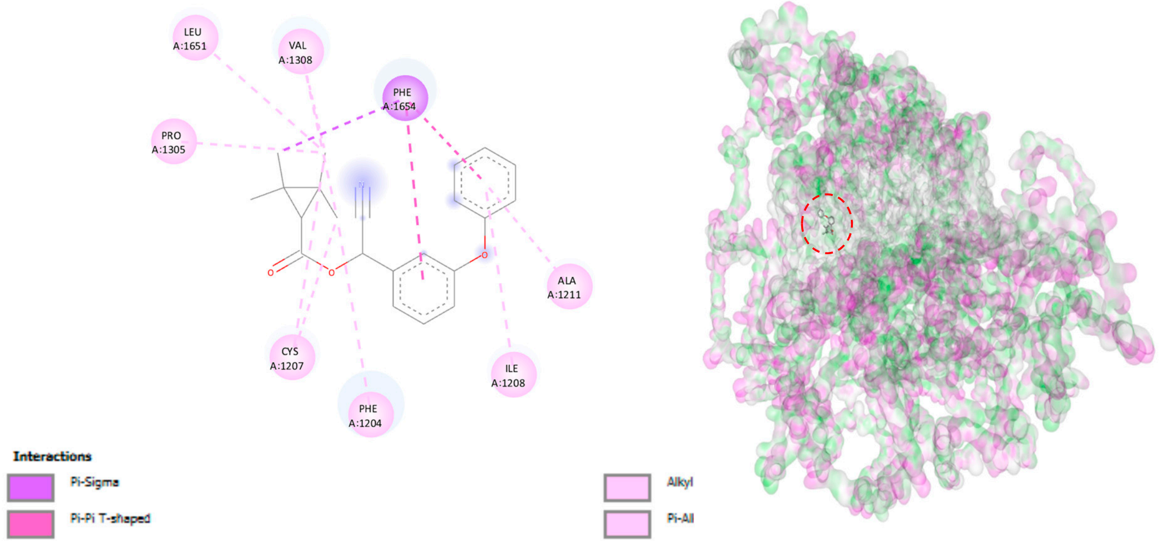
cacna1h	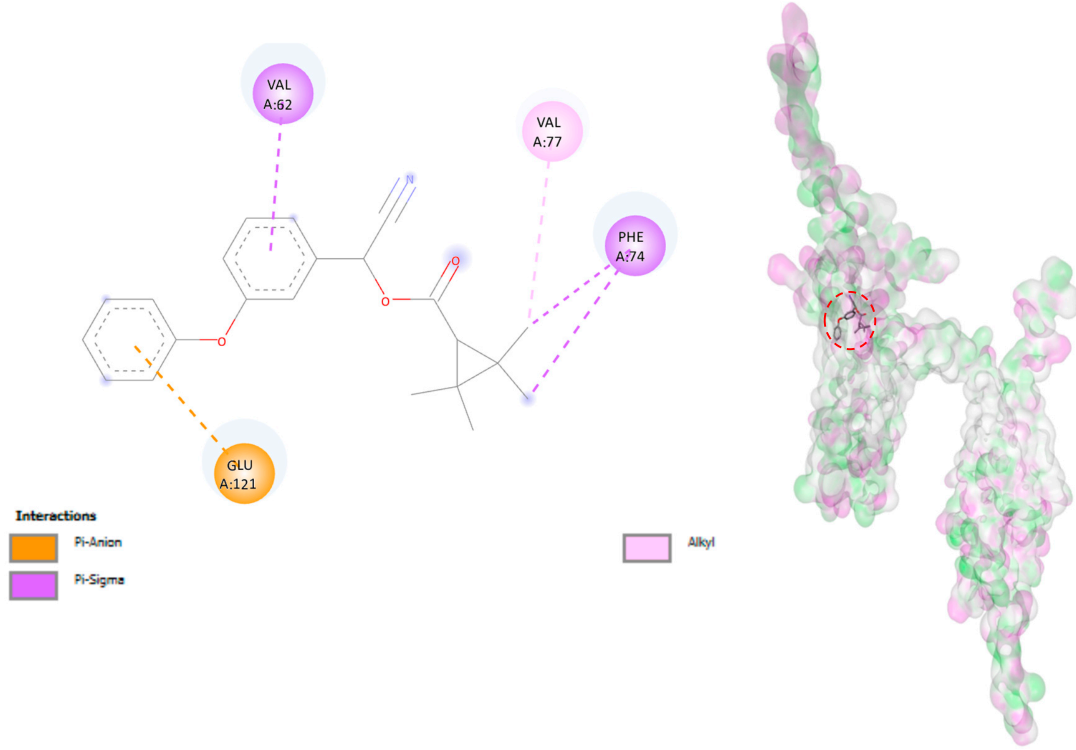

cacna1fa

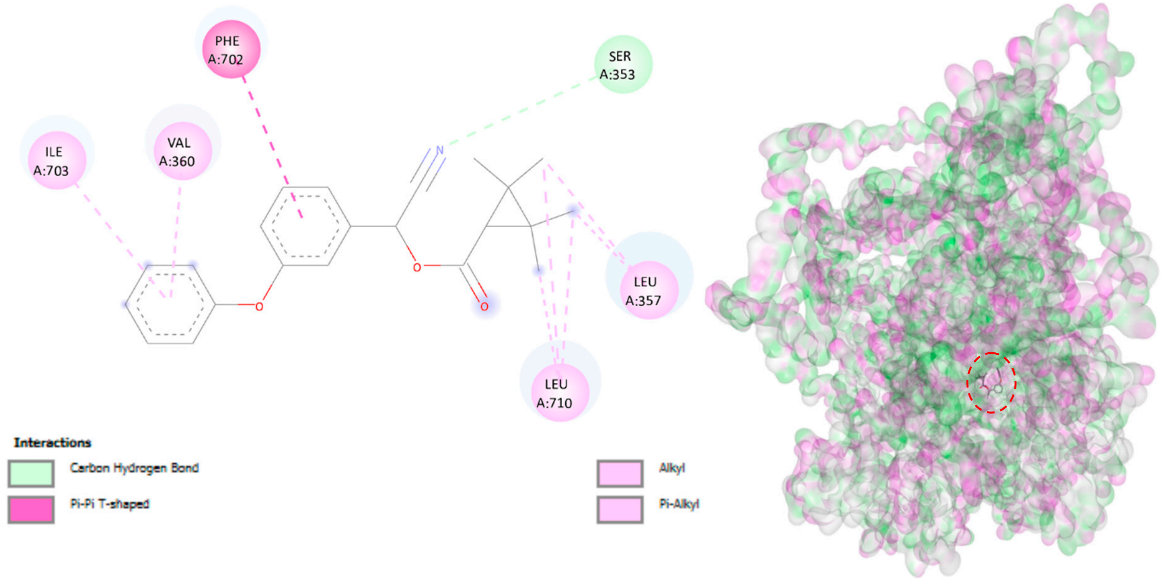

mdm2

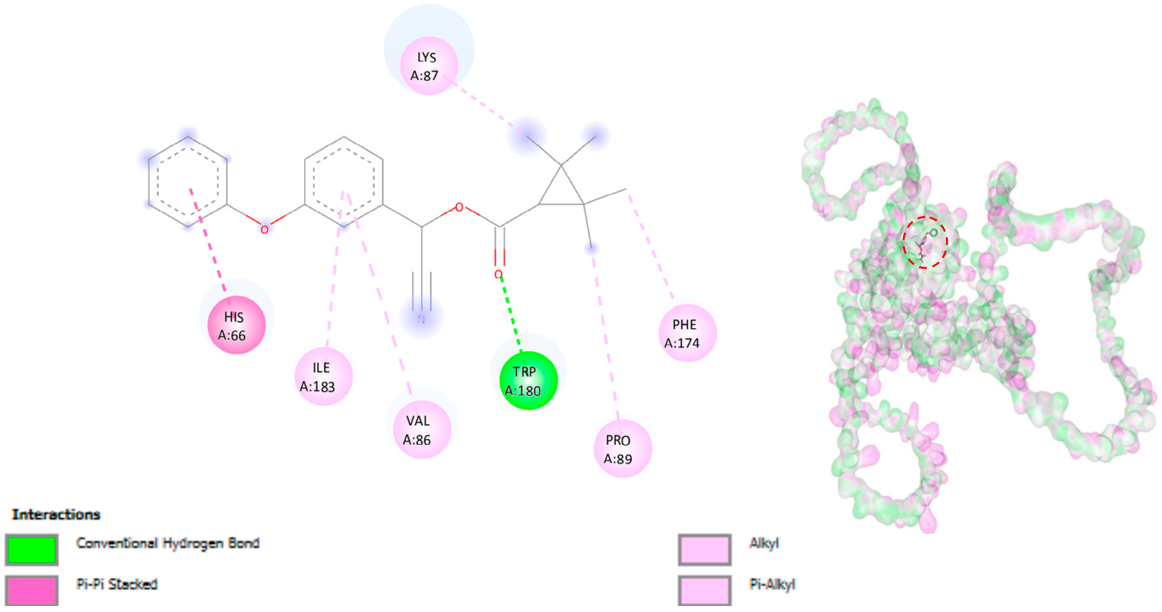

Supplement: Supplementary file 1 [file biology-12-01214-s001.zip › Supplementary Table.pdf]
